# Editorial: Global excellence in renal pharmacology 2022: Central and South America

**DOI:** 10.3389/fphar.2023.1362010

**Published:** 2024-01-09

**Authors:** Vinicius Andrade-Oliveira, Orestes Foresto-Neto, Niels Olsen Saraiva Câmara

**Affiliations:** ^1^ Bernardo’s Lab, Center for Natural and Human Sciences, Federal University of ABC, São Paulo, Brazil; ^2^ Nephrology Division, Universidade Federal de São Paulo, São Paulo, Brazil; ^3^ Laboratory of Immunobiology of Transplantation, Department of Immunology, Institute of Biomedical Sciences, Universidade de São Paulo, São Paulo, Brazil

**Keywords:** enteroendocine cells, antibiotic, renal pharmacology, inflammation, immunossuppression

Acute kidney injury (AKI) and chronic kidney disease (CKD) pose significant challenges to global public health. AKI is characterized by a swift and sudden decline in kidney function, exacerbating the outcomes of patients admitted to the intensive care unit. Various factors, including bacterial and viral infections, drug toxicity, and transplantation, have been linked to this condition. These factors instigate the activation of signaling pathways in renal and infiltrated cells, inducing inflammation and cell death in the renal parenchyma.

Immune cells, altered cell metabolism, cytokines, chemokines, gut dysbiosis, and bacterial or viral infections have been explored as potential targets and biomarkers for both AKI and CKD (Andrade-Oliveira et al., 2019; Basso et al., 2021; Gharaie et al., 2023; Andrade-Oliveira et al., 2015; Jang and Rabb, 2015; Andrade Silva et al., 2021; Foresto-Neto et al., 2018; Foresto-Neto et al., 2020; Felizardo et al., 2019; Watanabe et al., 2020). Notably, SARS-CoV-2 has been associated with the infection of renal cells, and COVID-19 has demonstrated a detrimental impact on patients with AKI and CKD (Diao et al., 2021; Arenas et al., 2023; Mackintosh et al., 2023).

The surge in bacterial infections has prompted a widespread global use of antibiotics, with the potential risk of nephrotoxicity associated with these medications. Therefore, a critical step in mitigating drug-induced acute kidney injury (AKI) is to evaluate the impact of antibiotics on renal function.

Addressing this concern, (Baiocco et al.) demonstrated the efficacy of implementing an institutional protocol for the safe administration of vancomycin. This protocol, encompassing appropriate dosing, timely monitoring of serum levels, and effective communication of critical results, facilitated the early detection of toxic levels, thereby reducing the risk of AKI. In a retrospective study involving 211 patients, they observed that those admitted post-protocol implementation exhibited a lower frequency of AKI (20.9%) compared to patients pre-protocol (38.4%). This highlights the significant benefits of the judicious use of vancomycin for patients.

In a prospective study involving 94 patients, (Sampaio de Souza Garms et al.) delved deeper into vancomycin-induced acute kidney injury (AKI). They identified a frequency of 24.5%, manifesting approximately 11 days after the initial vancomycin administration, with KDIGO stage 1 (Khwaja, 2012) being the most prevalent (61%). Factors such as age, plasmatic vancomycin concentration, and NGAL levels between 96 and 144 h after the first day of vancomycin use were pinpointed as contributors to vancomycin-induced AKI. Intriguingly, the urinary biomarker TIMP-2 multiplied by IGFBP7 between 144 and 192 h, within the vancomycin use period, and higher plasmatic vancomycin concentrations between 192 and 240 h were associated with the non-recovery of kidney function at the time of hospital discharge. These studies offer valuable insights into potential pathways and markers linked to kidney injury induced by vancomycin administration.

Rapamycin, an immunosuppressive drug widely utilized in transplantation settings, targets the serine/threonine kinase activity of the mammalian target of rapamycin (mTOR). However, its use is limited due to adverse effects. In this regard, (Peres et al.) demonstrated that BALB/c animals receiving rapamycin exhibited increased proteinuria compared to non-treated animals. The researchers observed that rapamycin reduced tubular protein uptake by decreasing megalin expression in the brush border membrane and disrupting its subcellular distribution in proximal tubular epithelial cells. Remarkably, this effect occurred without alterations in the glomerular structure and function. This suggests that the elevated proteinuria associated with rapamycin use may originate in the tubular compartment.

The kidneys are influenced by factors from other organs, and elements originating from the gut, such as microbial metabolites, can impact the outcome of acute kidney injury (AKI) and chronic kidney disease (CKD) (Foresto-Neto et al., 2021). Conversely, kidney diseases can alter the gut microbiota, contributing to kidney damage and establishing a vicious cycle (Felizardo et al., 2016). Therefore, comprehending the factors associated with the kidney-gut axis can aid in identifying new targets for treating kidney diseases. In this context, (Nery Neto et al.) demonstrated that enteroendocrine cells, a subset of specialized gut epithelial cells that secrete hormones upon stimulation, may represent a missing link in the gut and kidney crosstalk. Kidney cells express hormone receptors, making them susceptible to the hormones produced by enteroendocrine cells. Through *in silico* analyses of publicly available datasets, the researchers reported that enteroendocrine hormone receptors are differentially expressed in various models of AKI and CKD.

In essence, these studies contribute valuable insights into the intricate mechanisms underlying kidney health and disease, emphasizing the importance of targeted interventions, careful medication management, and a holistic understanding of the interorgan relationships involved in renal function. Such knowledge holds the promise of advancing both preventive and therapeutic strategies for acute and chronic kidney conditions.
